# Strong Association between Plasma Dipeptidyl Peptidase-4 Activity and Impaired Cognitive Function in Elderly Population with Normal Glucose Tolerance

**DOI:** 10.3389/fnagi.2017.00247

**Published:** 2017-07-26

**Authors:** Bo Chen, Tianpeng Zheng, Linyuan Qin, Xueping Hu, Xiaoxi Zhang, Yihong Liu, Hongbo Liu, Shenghua Qin, Gang Li, Qinghua Li

**Affiliations:** ^1^Department of Human Anatomy, Southwest Medical University Luzhou, China; ^2^Research Center of Combine Traditional Chinese and Western Medicine, Affiliated Traditional Medicine Hospital of Southwest Medical University Luzhou, China; ^3^Department of Endocrinology and Metabolism, The Second Affiliated Hospital of Guilin Medical University Guilin, China; ^4^Center of Diabetic Systems Medicine, Guilin Medical University Guilin, China; ^5^Department of Epidemiology and Health Statistics, Guilin Medical University Guilin, China; ^6^Diabetic Centre of Control and Prevention, The People’s Liberation Army 520 Hospital Mianyang, China; ^7^Department of Laboratory Medicine, The Second Affiliated Hospital of Guilin Medical University Guilin, China; ^8^Medical Examination Center, Affiliated Hospital of Guilin Medical University Guilin, China; ^9^Department of Psychiatry, Affiliated Hospital of Guilin Medical University Guilin, China; ^10^Department of Neurology, Affiliated Hospital of Guilin Medical University Guilin, China

**Keywords:** dipeptidyl peptidase-4, impaired cognitive function, oxidative stress, inflammation, glucagon-like peptide-1, biomarker, therapeutic target

## Abstract

**Objective:** Inflammation, oxidative stress, and decreased glucagon-like peptide-1 (GLP-1) are risk factors for cognitive impairment. Dipeptidyl peptidase-4 (DPP4) was identified as a novel adipokine capable of enhancing these risk factors. Hence, we investigated the relationship between plasma DPP4 activity and impaired cognitive function in elderly Chinese population with normal glucose tolerance (NGT).

**Methods:** We performed a cross-sectional study using data from 1229 elderly participants (60 years or older) in Guilin. Plasma DPP4 activity, oxidative stress parameters, fasting active GLP-1, and inflammatory markers were measured in all participants. Impaired cognitive function was diagnosed according to the National Institute on Aging-Alzheimer’s Association workgroups criteria.

**Results:** Participants in the upper quartile of plasma DPP4 activity had higher C-reactive protein (CRP), interleukin-6 (IL-6), 8-iso-PGF2a, nitrotyrosine, and lower GLP-1 and Montreal Cognitive Assessment (MoCA) scores compared with those in the lowest quartile (*P* < 0.001). The odds ratios (ORs) for increased CRP, IL-6, 8-iso-PGF2a, nitrotyrosine, and decreased active GLP-1 were higher with increasing DPP4 quartiles after adjustment for confounders (all *P* < 0.001). In the highest DPP4 quartile, impaired cognitive function risk was higher (OR, 2.26; 95% confidence interval, 1.36–3.76) than in the lowest quartile after adjustment for potential confounders. The risk for impaired cognitive function increased more with higher levels of DPP4 activity, nitrotyrosine and 8-iso-PGF2a (*P* < 0.05), but not with higher IL-6, CRP or lower GLP-1.

**Conclusion:** Plasma DPP4 activity is significantly and independently associated with impaired cognitive function, mainly executive, in elderly Chinese population with NGT. The underlying mechanisms for this association may be partly attributed to the effect of DPP4 on oxidative stress. Plasma DPP4 activity might serve as a risk biomarker or therapeutic target for the prevention and treatment of impaired cognitive function.

## Introduction

Mild cognitive impairment is accepted as an intermediate stage between normal cognitive functioning and dementia (Petersen, 2004; Petersen et al., 2009). Once mild cognitive impairment becomes more advanced, interventions targeted at pathogenesis of dementia are unlikely to delay further cognitive impairment (Kaduszkiewicz et al., 2005; Salloway et al., 2009; Sperling et al., 2011; Zheng et al., 2016). Consequently, it is of great importance to identify potentially modifiable risk factors and therapeutic targets for impaired cognitive function in elderly population.

Accumulated evidence reveals that inflammation and oxidative stress may play critical roles in the pathogenesis of cognitive decline (Furney et al., 2011; Koyama et al., 2013; Biessels et al., 2014; Swomley and Butterfield, 2015). Moreover, emerging evidence supports the contribution of glucagon-like peptide-1 (GLP-1) to cognitive preservation (Kravitz et al., 2013). Dipeptidyl peptidase-4 (DPP4) is a widely expressed multifunctional exopeptidase that exists as a membrane-anchored cell surface protein or in a soluble form in the plasma (Matteucci and Giampietro, 2009). It has been identified as a novel adipokine playing crucial roles in GLP-1 degradation and in the development of inflammation and oxidative stress (Drucker and Nauck, 2006; Lamers et al., 2011; Zheng et al., 2015a,b). More interestingly, animal studies have proved that DPP4 activity inhibitors ameliorated cognitive impairment through suppressing inflammatory reaction, oxidative stress, or GLP-1 degradation (Gault et al., 2015; Ma et al., 2015; Tsai et al., 2015).

DPP4 activity inhibitors have now been widely used as antidiabetic drugs at clinical practice, however, no research has ever investigated the relationship between impaired cognitive function and plasma DPP4 activity in elderly Chinese population with normal glucose tolerance (NGT), or considered the feasibility of identifying plasma DPP4 activity as a biomarker or therapeutic target for the prevention and treatment of impaired cognitive function. Consequently, in this study, we investigated the relationship between impaired cognitive function and plasma DPP4 activities in a cross-sectional study of 1229 elderly Chinese population with NGT, since we aimed to further explore the underlying mechanisms and clinical implications for such relationship from a clinical perspective, the associations between plasma DPP4 activities and pathogenetic factors for impaired cognitive function mentioned above were evaluated as well.

## Materials and Methods

### Subjects

Because hyperglycemia may have a mutual effect with DPP4 and cognitive function (Biessels et al., 2014; Zheng T. et al., 2014), introducing an additional confounding factor into this study, the current study was performed in Chinese population with NGT. A total of 1229 elderly Chinese participants, aged 60–85 years, who had undergone routine health examinations at the Medical Examination Center of Affiliated Hospital of Guilin Medical University between 2013 and 2015 were recruited for analysis. All subjects visited the Examination Center for annual health examinations comprising screening tests for the detection of hypertension, hyperglycemia, malignancy, osteoporosis, cognitive impairment, etc. Inclusion criteria were: (1) elderly Chinese with NGT (≥60 years old); (2) long-term residence (≥5 years) in Guangxi Province; (3) being able to provide written informed consent. Participants meeting the following criteria were excluded: (1) those with any of the diseases comprising inflammatory diseases, autoimmune disease, hypothyroidism, malignancy, hypertensive crisis, dementia, head trauma, respiratory, heart, kidney, and liver dysfunction or failure. (2) Those with histories of central nervous system diseases that could lead to dementia (3) use of possible or known drugs affecting cognitive function or DPP4 activity for more than 3 months or at any time within 12 months before the enrollment. (4) Those with histories of auditory/visual disorders, psychological disturbances and severe hypoglycemia. (5) Drug or alcohol abuse or dependence (6) participants with incomplete data. The study has been approved by the Ethics Committee at Affiliated Hospital of Guilin Medical University, all participants gave written informed consent in accordance with the Declaration of Helsinki. This study was registered on the Chinese clinical trial registry (ChiCTR-EPC-14005273).

The purpose, nature, and potential risks of the experiments were fully explained to the subjects, and all subjects gave written informed consent at the beginning of the study. The subjects had the full capacity to consent because they maintained general cognitive function and daily activities. We included only patients who had been fully able to understand and cooperate with study procedures.

### Data Collection

All participants completed a standard questionnaire containing questions about demographic characteristics, life style risk factors, education level, annual income, cognitive function, the histories of present and past illness and medical therapy. Anthropometric parameters such as blood pressure, body weight, height, and body mass index were measured and calculated as previously described (Yang et al., 2010). Venous blood samples were collected after an overnight fast to measure biochemical values, inflammatory markers, oxidative parameters, active GLP-1, and DPP4 activity.

### Clinical and Laboratory Evaluations

Circulating levels of interleukin-6 (IL-6), C-reactive protein (CRP), 8-iso-PGF2a, nitrotyrosine, fasting active GLP-1, and DPP4 activity were measured as previously described (Zheng T. et al., 2014; Zheng et al., 2015a,b, 2016).

Impaired cognitive function was diagnosed according to the National Institute on Aging-Alzheimer’s Association workgroups criteria (Albert et al., 2011). The criteria were (1) cognitive complaint usually coming from the patients or their family, (2) impaired cognitive function in one or several domains [assessed in our study by Montreal Cognitive Assessment (MoCA)], (3) essentially preserving most activities of daily living (assessed in our study by basic and instrumental Activities of Daily Living questionnaires), (4) absence of dementia (assessed in our study by DSM-V criteria). The MoCA assesses multiple cognitive domains, including concentration and attention, executive function, memory, language ability, visual–spatial skills, conceptual thinking, calculation performance, and orientation. MoCA scores range from 0 to 30, the normal score is ≥26, with one point added for people with less than 12 years of education (Nasreddine et al., 2005). The final diagnosis of impaired cognitive function was further confirmed by a multidisciplinary team including neuropsychologists, psychiatrists, and neurologists (Zheng et al., 2016).

### Statistical Analysis

Statistical analyses were performed using SPSS version 16 statistical package (SPSS, Chicago, IL, United States). All continuous variables were expressed as means ± SD or medians (interquartile ranges), and all categorical variables were reported as frequency and percentage. Comparisons of means and proportions were performed with an analysis of covariance (CRP, IL-6, 8-iso-PGF2a, nitrotyrosine, fasting active GLP-1, MoCA score, and DPP4 activity), χ^2^ or *t*-test. Pearson’s correlation analyses and partial correlation analyses were used to examine the correlations between DPP4 activity and other variables. The multivariate logistic regression analyses were used to calculate odds ratios (ORs) for impaired cognitive function, elevated proinflammatory markers, oxidative stress parameters, and decreased fasting active GLP-1. Owing to a lack of normal range of CRP, IL-6, 8-iso-PGF2a, nitrotyrosine, and fasting active GLP-1 in elderly Chinese population, the highest quartiles of CRP, IL-6, 8-iso-PGF2a, and nitrotyrosine were defined as increased, whereas the lowest quartiles of GLP-1 were defined as decreased.

## Results

### Clinical and Laboratory Characteristics

**FIGURE 1:**
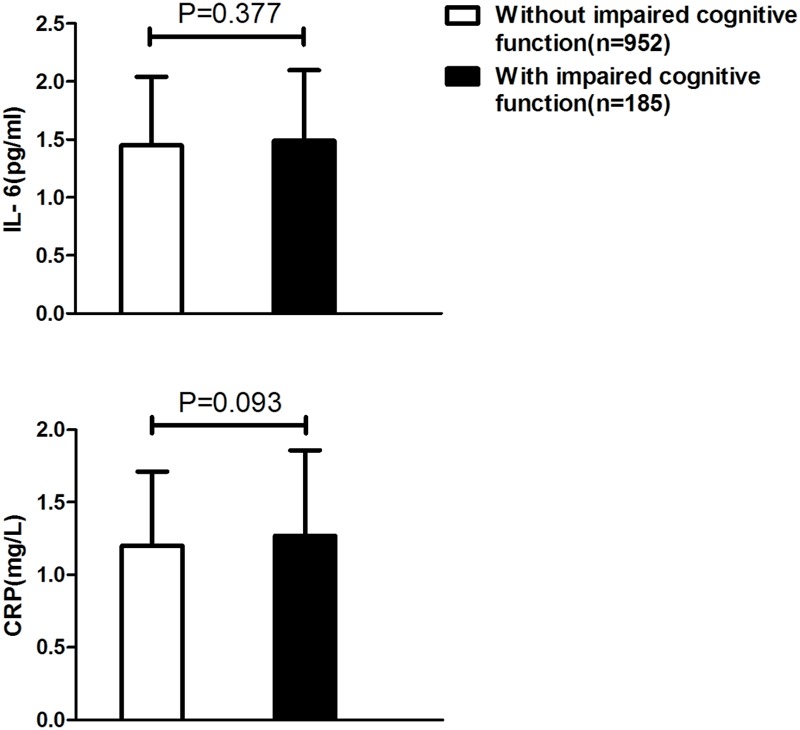
Comparisons of inflammation markers between participants with impaired cognitive function and those without impaired cognitive function after excluding subjects with cardiovascular diseases.

**Table 1** showed the demographic characteristics and laboratory data of participants according to DPP4 activity quartiles. Patients with higher DPP4 activities tended to be relatively old (*P* = 0.006) with high levels of BMI, TG, IL-6, CRP, nitrotyrosine, and 8-iso-PGF2a (all *P* < 0.001), and lower fasting active GLP-1 and MoCA score (*P* < 0.001); **Table 2** showed that participants with impaired cognitive function had higher nitrotyrosine, 8-iso-PGF2a, DPP4 activity, and lower MoCA score compared to those without impaired cognitive function. IL-6, CRP, and fasting active GLP-1 did not differ between two groups. After excluding participants with cardiovascular disease, IL-6 and CRP levels were still not found elevated in impaired cognitive function patients compared to healthy controls (**Figure 1**).

**Table 1:** Characteristics of study participants according to DPP4 activity quartiles.

Characteristics	Total (*n* = 1229)	Q1 (*n* = 307)	Q2 (*n* = 309)	Q3 (*n* = 306)	Q4 (*n* = 307)	*P*-value
		<11.8	11.8–16.9	17.0–24.0	>24.0	
DPP4 activity(nmol/min/mL)	18.1 ± 8.2	8.5 ± 2.1	14.4 ± 1.5	20.0 ± 2.1	29.4 ± 4.7	<0.001
Age (years)	69.2 ± 5.6	68.6 ± 5.4	69.3 ± 5.6	68.7 ± 5.3	70.0 ± 5.8	0.006
Percent men (%)	47.5	53.1	43.0	45.4	48.5	0.073
Body mass index (kg/m^2^)	23.3 ± 3.7	22.5 ± 3.5	23.1 ± 3.6	23.6 ± 3.7	23.8 ± 3.8	<0.001
Current smoking (%)	21.1	19.2	17.5	22.5	25.1	0.093
Habitual alcohol drinking (%)	18.4	16.9	14.9	21.6	20.2	0.128
Leisure-time physical activity (%)	56.6	59.0	60.2	56.9	50.2	0.057
**Education level**						0.158
≤Primary school	49.9	48.2	49.5	49.3	52.4	
Middle school	41.5	41.0	45.3	40.2	39.4	
≥High school	8.6	10.7	5.2	10.5	8.1	
**Annual income, RMB**						0.246
≤5000	5.3	5.2	3.9	5.9	6.2	
5000–30,000	45.9	46.3	45.6	50.7	41.0	
>30,000	48.8	48.5	50.5	43.5	52.8	
Cardiovascular disease (%)	7.5	5.9	6.5	9.2	8.5	0.351
Statin use (%)	11.4	8.5	14.9	12.1	10.1	0.072
NSAID use (%)	5.7	4.2	6.1	7.2	5.2	0.433
SBP^a^ (mmHg)	121 ± 19	120 ± 16	119 ± 16	122 ± 19	122 ± 24	0.213
DBP^a^ (mmHg)	71 ± 9	71 ± 8	71 ± 9	72 ± 8	70 ± 11	0.102
TG (mmol/L)^a^	1.44 (1.09–1.97)	1.14 (0.85–1.70)	1.41 (1.10–2.06)	1.50 (1.19–2.06)	1.61 (1.25–2.12)	<0.001
TC (mmol/L)^a^	5.12 ± 0.95	5.01 ± 0.84	5.10 ± 0.97	5.19 ± 0.93	5.17 ± 1.04	0.346
LDL-C (mmol/L)^a^	3.04 ± 0.90	2.98 ± 0.95	3.08 ± 0.83	3.05 ± 0.89	3.04 ± 0.92	0.890
HDL-C (mmol/L)^a^	1.40 ± 0.38	1.41 ± 0.39	1.38 ± 0.38	1.43 ± 0.37	1.37 ± 0.36	0.215
IL-6 (pg/mL)^a^	1.36 (1.11–1.61)	1.30 (1.10–1.49)	1.27 (0.86–1.45)	1.39 (1.22–1.71)	1.56 (1.23–1.92)	<0.001
CRP(mg/L)^a^	1.15 (0.93–1.35)	1.06 (0.79–1.24)	1.15 (0.90–1.27)	1.16 (0.99–1.33)	1.30 (0.98–1.59)	<0.001
Nitrotyrosine (μmol/L)^a^	0.39 ± 0.12	0.36 ± 0.11	0.35 ± 0.09	0.41 ± 0.12	0.45 ± 0.13	<0.001
8-iso-PGF2a (pg/mL)^a^	42.4 ± 10.8	37.7 ± 10.2	43.4 ± 8.2	40.2 ± 12.2	48.1 ± 9.1	<0.001
Fasting active GLP-1 (pmol/L)^a^	3.31 ± 1.28	3.56 ± 1.41	3.41 ± 1.38	3.11 ± 1.06	3.15 ± 1.19	<0.001
MoCA score^a^	26.8 ± 2.4	27.7 ± 2.0	27.4 ± 2.3	26.4 ± 2.2	25.9 ± 2.6	<0.001

*Data were expressed as means ± standard deviation, median (interquartile range), or percentage for normally distributed continuous various, abnormally distributed continuous variables (TG, IL-6, CRP), and categorical variables, respectively. Cigarette smoking was defined as having smoked at least 100 cigarettes in one’s lifetime. Regular leisure-time physical activity was defined as participation in ≥30 min of moderate or vigorous activity per day at least 3 days per week. ^a^Adjusted for age, gender, and BMI.*

**Table 2:** Characteristics of the 1229 participants by impaired cognitive function.

	Without impaired cognitive function	With impaired cognitive function	*P*-value
*n*	1026	203	-
Age (years)	68.8 ± 5.5	70.8 ± 5.6	<0.001
Percent men (%)	46.6	52.2	0.142
BMI (kg/m^2^)	23.1 ± 3.6	23.9 ± 3.9	0.010
Current smoking (%)	20.8	22.7	0.544
Habitual alcohol drinking (%)	18.0	20.2	0.467
Leisure-time physical activity (%)	56.8	55.2	0.665
Education level			0.002
≤Primary school	47.9	60.1	
Middle school	42.7	35.5	
≥High school	9.5	4.4	
Annual income, RMB			0.813
≤5000	5.3	5.4	
5000–30,000	46.3	43.8	
>30,000	48.4	50.7	
Cardiovascular disease (%)	7.2	8.9	0.413
Statin use (%)	10.5	15.8	0.032
NSAID use (%)	5.6	6.4	0.634
IL-6 (pg/mL)^a^	1.35 (1.11–1.58)	1.39 (1.10–1.73)	0.087
CRP (mg/L)^a^	1.15 (0.93–1.34)	1.17 (0.95–1.46)	0.161
Nitrotyrosine (μmol/L)^a^	0.38 ± 0.12	0.43 ± 0.14	<0.001
8-iso-PGF2a (pg/mL)^a^	41.6 ± 10.4	46.4 ± 11.5	<0.001
Fasting active GLP-1 (pmol/L)^a^	3.33 ± 1.29	3.22 ± 1.24	0.324
MoCA score^a^	27.7 ± 1.3	22.4 ± 1.8	<0.001
DPP4 activity (nmol/min/mL)^a^	17.5 ± 8.1	20.9 ± 8.4	<0.001

*^a^Adjusted for age, gender, and BMI.*

### Relationship between Plasma DPP4 Activity and Pathogenetic Factors for Impaired Cognitive Function

Correlation analysis showed that plasma DPP4 activities were positively and significantly related to age (*r* = 0.074, *P* = 0.009), BMI (*r* = 0.130, *P* < 0.001), IL-6 (*r* = 0.305, *P* < 0.001), CRP (*r* = 0.243, *P* < 0.001), nitrotyrosine (*r* = 0.294, *P* < 0.001), 8-iso-PGF2a (*r* = 0.333, *P* < 0.001) and negatively with fasting active GLP-1 (*r* = -0.146, *P* < 0.001) and MoCA score (*r* = -0.299, *P* < 0.001). After adjustments for age, gender, BMI, and education level, these associations still remained statistically significant (**Table 3** and **Supplementary Figure S1**).

**Table 3:** Correlations between DPP4 activities vs metabolic parameters and MoCA score.

	DPP4 activity^a^		DPP4 activity^b^	
	*r*	*P*	*r*	*P*
Age	0.074	0.009	–	–
BMI	0.130	<0.001	–	–
IL-6	0.305	<0.001	0.306	<0.001
CRP	0.243	<0.001	0.226	<0.001
Nitrotyrosine	0.294	<0.001	0.289	<0.001
8-iso-PGF2a	0.333	<0.001	0.327	<0.001
Fasting active GLP-1	-0.146	<0.001	-0.142	<0.001
MoCA score	-0.299	<0.001	-0.283	<0.001

*^a^*P*-value determined by Pearson’s correlation analysis with respect to the DPP4 activity.*

*^b^*P*-value determined by partial correlation analysis with respect to the DPP4 activity adjusted for age, BMI, gender, and education level.*

As shown in **Table 4**, the upper plasma DPP4 activities quartile had higher ORs for increased CRP, IL-6, nitrotyrosine, 8-iso-PGF2a and decreased active GLP-1 compared to the lowest one after adjustment for potential confounders. In the upper quartile of plasma DPP4 activities, the ORs were 3.56 [95% confidence interval (CI) 2.39–5.31] for elevated IL-6, 4.31 (2.86–6.49) for elevated CRP, 5.19 (3.45–7.82) for elevated nitrotyrosine, 6.39 (4.06–10.08) for elevated 8-iso-PGF2a, and 3.22 (2.17–4.77) for decreased active GLP-1 after adjustments for possible confounders.

**Table 4:** Adjusted ORs and 95% CIs for increased inflammation, oxidative stress, and decreased fasting active GLP-1 according to DPP4 quartiles.

	DPP4 activity			
	Q1	Q2	Q3	Q4
**Elevated IL-6**				
Model 1	1.0	0.76 (0.48–1.20) 0.237	2.09 (1.41–3.10) <0.001	3.67 (2.51–5.36) <0.001
Model 2	1.0	0.76 (0.48–1.21) 0.244	2.18 (1.45–3.29) <0.001	3.56 (2.39–5.31) <0.001
Elevated CRP				
Model 1	1.0	1.28 (0.82–1.99) 0.278	1.84 (1.21–2.80) 0.005	4.82 (3.25–7.17) <0.001
Model 2	1.0	1.19 (0.76–1.87) 0.450	1.64 (1.06–2.52) 0.026	4.31 (2.86–6.49) <0.001
**Elevated nitrotyrosine**				
Model 1	1.0	0.80 (0.49–1.30) 0.373	2.66 (1.76–4.01) <0.001	5.30 (3.56–7.89) <0.001
Model 2	1.0	0.79 (0.48–1.28) 0.332	2.65 (1.74–4.03) <0.001	5.19 (3.45–7.82) <0.001
Elevated 8-iso-PGF2a				
Model 1	1.0	2.41 (1.50–3.86) <0.001	4.19 (2.66–6.59) <0.001	6.32 (4.05–9.87) <0.001
Model 2	1.0	2.39 (1.48–3.84) <0.001	4.12 (2.60–6.52) <0.001	6.39 (4.06–10.08) <0.001
**Decreased active GLP-1**				
Model 1	1.0	1.22 (0.80–1.85) 0.358	3.31 (2.25–4.84) <0.001	3.15 (2.15–4.63) <0.001
Model 2	1.0	1.19 (0.78–1.82) 0.419	3.40 (2.30–5.02) <0.001	3.22 (2.17–4.77) <0.001

*Model 1: crude model.*

*Model 2: model 1 *+* age *+* gender *+* BMI *+* leisure-time physical activity *+* cardiovascular disease *+* SBP *+* TG.*

### Relationship between Plasma DPP4 Activity and Impaired Cognitive Function

Among the 1229 elderly Chinese participants included in this study, 203 patients (16.5%) had impaired cognitive function. The impaired cognitive function prevalence according to plasma DPP4 activity quartiles were 9.1, 16.5, 15.0, and 25.4%, respectively.

Multivariate logistic regression analysis showed that the ORs for impaired cognitive function were significantly higher in the upper quartile of plasma DPP4 activities than in the lower quartile. The OR was 3.15 (1.94–5.12) for impaired cognitive function after adjustments for confounders. Interestingly, further adjustment for 8-iso-PGF2a reduced the magnitude of the OR for impaired cognitive function [2.26 (1.36–3.76), *P* = 0.002], however, this relationship was not attenuated after further adjustments for IL-6 or fasting active GLP-1 (**Table 5**). This independent relationship between DPP4 activity and impaired cognitive function still existed in a subgroup of participants without hyperuricemia and hypertriglyceridemia (Supplementary Table S1).

**Table 5:** Logistic regression analysis of the association of DPP4 activity and impaired cognitive function.

	DPP4 activity			
	Q1	Q2	Q3	Q4
DPP4 activity (nmol/mL/min)	<11.8	11.8–16.9	17.0–24.0	>24.0
Impaired cognitive function	28 (9.1%)	51 (16.5%)	46 (15.0%)	78 (25.4%)
Model 1	1	1.97 (1.21–3.22) 0.007	1.76 (1.07–2.90) 0.026	3.39 (2.13–5.41) <0.001
Model 2	1	1.85 (1.12–3.06) 0.017	1.78 (1.06–2.97) 0.028	3.15 (1.94–5.12) <0.001
Model 3	1	1.81 (1.09–3.01) 0.022	1.82 (1.09–3.05) 0.023	3.31 (2.02–5.42) <0.001
Model 4	1	1.85 (1.11–3.06) 0.017	1.76 (1.05–2.95) 0.032	3.13 (1.92–5.08) <0.001
Model 5	1	1.57 (0.94–2.62) 0.084	1.67 (0.99–2.80) 0.054	2.26 (1.36–3.76) 0.002

*Model 1: crude model.*

*Model 2: model 1 *+* age *+* gender *+* BMI *+* current smoking *+* habitual alcohol consumption *+* leisure-time physical activity *+* education level *+* annual income *+* cardiovascular disease *+* statin use *+* NSAID use *+* SBP *+* TG *+* HDL-C.*

*Model 3: model 2 *+* IL-6.*

*Model 4: model 2 *+* fasting active GLP-1.*

*Model 5: model 2 *+* 8-iso-PGF2a.*

**FIGURE 2:**
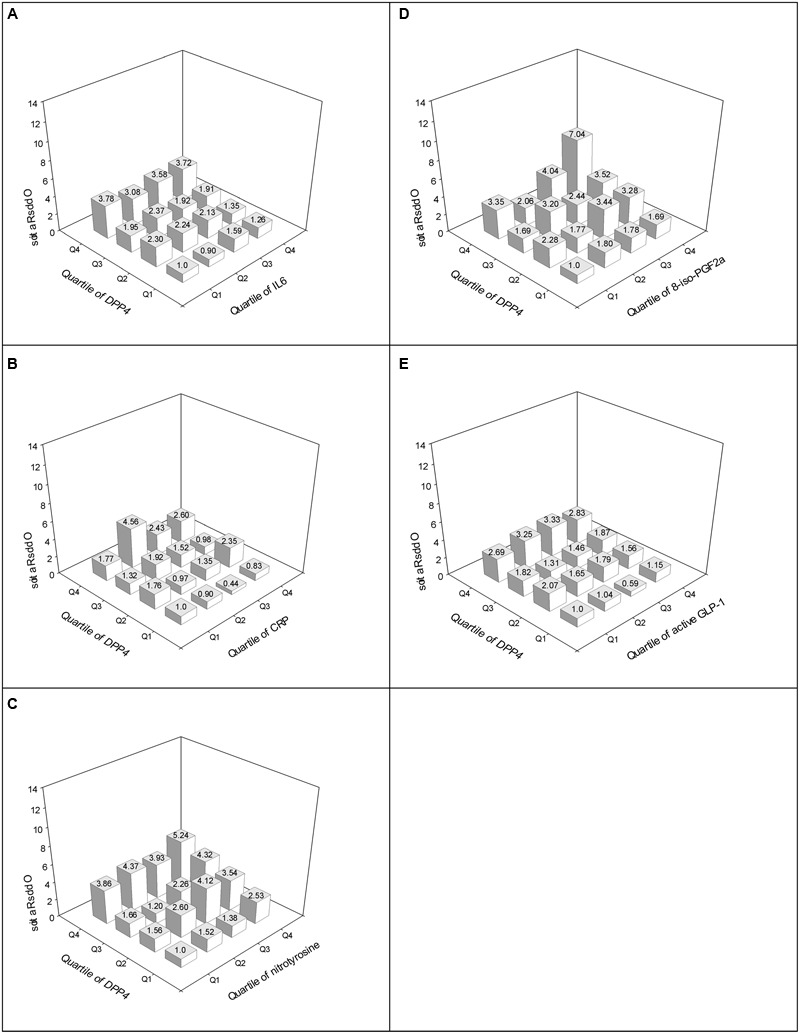
Adjusted ORs for impaired cognitive function according to the quartiles of DPP4 activity and IL-6 **(A)**, DPP4 activity and CRP **(B)**, DPP4 activity and nitrotyrosine **(C)**, DPP4 activity and 8-iso-PGF2a **(D)**, and DPP4 activity and fasting active GLP-1 **(E)**. Adjusted for age, gender, BMI, current smoking, habitual alcohol consumption, leisure-time physical activity, education level, annual income, cardiovascular disease, statin use, NSAID use, SBP, TG, HDL-C.

The ORs for impaired cognitive function became more pronounced among subjects with both increased plasma DPP4 activities and higher circulating levels of nitrotyrosine (**Figure 2C**) and 8-iso-PGF2a (**Figure 2D**), however, this increasing trend of impaired cognitive function risk were not observed in higher levels of IL-6 (**Figure 2A**), CRP (**Figure 2B**), and lower levels of fasting active GLP-1 (**Figure 2E**). Even in the lowest quartiles of nitrotyrosine and 8-iso-PGF2a, the risks for impaired cognitive function were 3.35- to 3.86-fold higher in the highest DPP4 quartile than in the lowest quartile (**Figure 2**).

## Discussion

In this cross-sectional study, several key findings emerged from our analysis assessing the relationship between DPP4 activity and impaired cognitive function in elderly Chinese population. Our data indicated that (1) elevated plasma DPP4 activities were positively related to impaired cognitive function and negatively related to MoCA score in elderly Chinese population with NGT; (2) this relationship was paralleled by an increase in circulating oxidative stress parameters; (3) increased levels of proinflammatory markers and decreased levels of fasting active GLP-1 in peripheral circulation were not associated with an increased risk of impaired cognitive function.

Previous findings have shown higher peripheral levels of inflammatory markers such as IL-6, CRP, TNF-α in patients with dementia compared to controls (Bermejo et al., 2008; Furney et al., 2011). Since impaired cognitive function has been suggested to be a prodromal stage of dementia with similar underlying mechanisms, higher levels of inflammation markers were also expected in patients with impaired cognitive function compared to controls, however, our data did not find support for the elevation of peripheral inflammatory markers such as IL-6 and CRP in impaired cognitive function. Our results are consistent with a previously meta-analysis, which indicated that no significant differences in inflammatory factors studied (i.e., IL-6, IL-10, tumor necrosis factor-α, CRP, etc.) were found between subjects with impaired cognitive function and healthy controls (Saleem et al., 2015). The reasons for the non-significant differences in inflammation markers could be summarized as follows. First, it might results from variability in diagnosis of impaired cognitive function and assay procedures for inflammatory cytokines to some extent. Second, peripheral inflammatory markers may not be representative of inflammatory activity within the CNS (Tarkowski et al., 2003; Kim et al., 2011). Third, patients with low-grade chronic inflammatory diseases such as cardiovascular disease or hyperglycemia were not excluded specifically in most previous studies, which may contributed to overlap between participants with and those without impaired cognitive function (Koyama et al., 2013; Saleem et al., 2015). However, even after excluding participants with cardiovascular disease and diabetes in our study, peripheral inflammatory markers were still not found elevated in impaired cognitive function patients compared to healthy controls. Finally, systemic inflammation may be a later event in the pathophysiological cascade of cognitive decline (Stephan et al., 2012; Saleem et al., 2015). The proinflammatory effects of DPP4 and its mechanisms have well been established by previous researches (Wronkowitz et al., 2014; Zheng T.P. et al., 2014), in this study, a positive relationship between systemic inflammation and DPP4 activity was also found in elderly Chinese population, IL-6 and CRP levels were positively related to DPP4 activities and increased across DPP4 quartiles, however, we did not find any association between increased risk of impaired cognitive function and higher levels of inflammatory markers. The ORs for impaired cognitive function according to plasma DPP4 activities quartiles were not further reduced after adjusting for IL-6. Collectively, it may therefore be hypothesized that the positive relationship between DPP4 activity and impaired cognitive function risk might not be explained by the effect of DPP4 on inflammation.

Aside from inflammation, previous studies have provided a wealth of information in regard to the pathogenetic role of oxidative stress in the development of cognitive decline, even at the stage of impaired cognitive function, Swomley and Butterfield (2015) reported that oxidative stress-induced damage to key proteins led to deficiencies in systems (e.g., energy metabolism, cell signaling, neurotransmitter release, proteasome) important to the brain, which might led to the progression of cognitive decline. Consistent with other clinical studies (Cervellati et al., 2013; Lopez et al., 2013), our data indicated that oxidative parameters such as nitrotyrosine and 8-iso-PGF2a in the periphery circulation were also found higher in patients with impaired cognitive function than those without, in addition, the ORs for impaired cognitive function were higher with increasing 8-iso-PGF2a and nitrotyrosine quartiles, these findings above support the hypothesis that oxidative stress might represents a sign of dementia pathology and could be an early event in the progression of impaired cognitive function to dementia. The pathogenetic role of DPP4 in oxidative stress has been well addressed by Ishibashi et al. (2013), in their study, DPP4 dose-dependently increased ROS generation in endothelial cells. Consistently, in our study, correlation analysis also supported a significant and positive relationship between DPP4 activity and oxidative parameters after adjustment for possible confounders, the ORs for increased circulating levels of nitrotyrosine and 8-iso-PGF2a gradually increased across plasma DPP4 activity quartiles, furthermore, the risk of impaired cognitive function became more pronounced among participants with rising plasma DPP4 activity and higher levels of oxidative stress parameters. Consequently, it may therefore be hypothesized that DPP4 may also promote impaired cognitive function development by increasing ROS generation and activating oxidative stress.

To examine the robustness of this relationship between DPP4 activity and impaired cognitive function and to obtain information on potential mechanisms that could mediate the observed relationship, we performed a set of logistic regression models comparing risk of impaired cognitive function between participants in the lowest DPP4 activity group and in highest DPP4 activity group. Interestingly, further adjustment for 8-iso-PGF2a yielded only a reduction of the impaired cognitive function risk across the DPP4 activity quartiles. Even within the lowest nitrotyrosine and 8-iso-PGF2a quartiles, the risks for impaired cognitive function were still 3.35- to 3.86-fold higher in the highest DPP4 quartile than in the lowest quartile. Thus, the positive relationship between increased plasma DPP4 activities and high impaired cognitive function risk in this non-diabetic Chinese population might not be merely the results of enhanced oxidative stress. Because of the pleiotropic effects of DPP4, the interactions between plasma DPP4 activities and other pathogenetic factors might promote the development of impaired cognitive function as well.

The neuroprotective actions of GLP-1, an incretin hormone secreted from L cells located in the distal ileum and colon, have been demonstrated in *in vivo* and *in vitro* studies. It has been proved to reduce apoptosis and inflammation, protect neurons from oxidative stress, protect synaptic plasticity and memory formation from the detrimental effects of Aβ, and enhance neurogenesis in the brains (Holscher, 2010; Kravitz et al., 2013). In this study, although a significant and inverse relationship was found between DPP4 activity and fasting active GLP-1 level, we failed to find a significant difference in fasting active GLP-1 levels between participants with impaired cognitive function and those without impaired cognitive function. The ORs for impaired cognitive function in the highest quartile of plasma DPP4 activities was not reduced after further adjusting for fasting active GLP-1. The reasons for this discrepancy could be summarized as follows. First, it is generally accepted that postprandial GLP-1 levels are much higher than fasting levels, it might be more appropriate to evaluate the association between impaired cognitive function and incretin hormones using postprandial GLP-1 rather than fasting GLP-1. However, this hypothesis remains to be validated because there is no direct clinical evidence indicating that GLP-1 contribution to impaired cognitive function is mainly postprandial. Second, the neuroprotective effects of GLP-1 were mostly observed in cell or animal studies, whereas this study was conducted in elderly Chinese population. The protective effect of GLP-1 for cognitive function might differ in various species. Third, although GLP-1 has been shown to cross the blood–brain barrier and increase brain GLP-1 concentrations (Hunter and Holscher, 2012), peripheral GLP-1 might not be able to reflect GLP-1 in the brain. Finally, the sample size, age, race, heterogeneity of impaired cognitive function and different assessments of cognitive impairment may lead to this discrepancy to some extent.

Because our study is cross-sectional, we cannot draw a causal conclusion that increased DPP4 activities promote the development of impaired cognitive function through its effect on oxidative stress. Although no direct evidence completed to date links impaired cognitive function back to the regulation of DPP4 activity, the parallel increase in oxidative stress and DPP4 activity could be interpreted in an opposite way.

## Limitations

Our study has several limitations. One limitation is the cross-sectional nature of present study. We could not prove a causal association between plasma DPP4 activities and the development of impaired cognitive function. Second, postprandial GLP-1 levels and its relationship with impaired cognitive function were not evaluated in this study. Third, Specific cognitive domains, such as episodic memory, cannot be evaluated by MoCA. Finally, because of the sample size and epidemiological nature of this study, neuropsychological assessment and radiological survey cannot be conducted to evaluate the specific reasons leading to impaired cognitive function, such as Alzheimer’s disease, vascular dementia, or Parkinson’s disease.

## Conclusion

In summary, our study demonstrated a positive and significant relationship between plasma DPP4 activities and impaired cognitive function in elderly non-diabetic population, the underlying mechanisms for this relationship may be partly attributed to the enhancement of oxidative stress promoted by DPP4. DPP4 inhibitors have now been wildly used as antidiabetic drugs at clinical practice, moreover, protective effects of DPP4 inhibitors on cognitive impairment have also been reported in a retrospective longitudinal study in patients with hyperglycemia (Rizzo et al., 2014). However, there have been no studies examining the safety and effectiveness of DPP4 inhibitor for the treatment of impaired cognitive function patients with NGT. Considering the fact that DPP4 activity inhibitors does not cause hypoglycemia, our findings in this study showed that DPP4 inhibitors might also hold promise for the treatment of impaired cognitive function in non-diabetic population but further controlled studies with specific clinical endpoints are still needed to assess their effects in this population. Identifying circulating DPP4 activity as a risk biomarker or therapeutic target for impaired cognitive function prevention and treatment in non-diabetic population may propose an interesting and important direction for further researches in this regard.

## Author Contributions

TZ, BC, XH, and QL contributed to study concept and design. TZ, LQ, BC, XH, XZ, YL, HL, SQ, GL, and QL contributed to acquisition, analysis, or interpretation of data. TZ, BC, and XH performed drafting the work. TZ, LQ, BC, XZ, YL, HL, SQ, GL, and QL critically revised the manuscript for important intellectual content. LQ performed statistical analysis. TZ obtained funding. TZ supervised the study. All authors approved the final manuscript to be published and agreed to be accountable for all aspects of the work in ensuring that questions related to the accuracy or integrity of any part of the work are appropriately investigated and resolved.

## Conflict of Interest Statement

The authors declare that the research was conducted in the absence of any commercial or financial relationships that could be construed as a potential conflict of interest.

## Supplementary Material

FIGURE S1 Correlations between DPP4 activities vs metabolic parameters and MoCA score represented by scatter plot. **(A)** relationship between DPP4 activity and IL-6; **(B)** relationship between DPP4 activity and CRP; **(C)** relationship between DPP4 activity and nitrotyrosine; **(D)** relationship between DPP4 activity and 8-iso-PGF2a; **(E)** relationship between DPP4 activity and fasting active GLP-1; **(F)** relationship between DPP4 activity and MoCA score.
