# Supplementary figures and images for: Strong Association between Plasma Dipeptidyl Peptidase-4 Activity and Impaired Cognitive Function in Elderly Population with Normal Glucose Tolerance
Source: Front Aging Neurosci. 2017 Jul 26;9:247. doi: 10.3389/fnagi.2017.00247 (PMC5526854; doi:10.3389/fnagi.2017.00247)

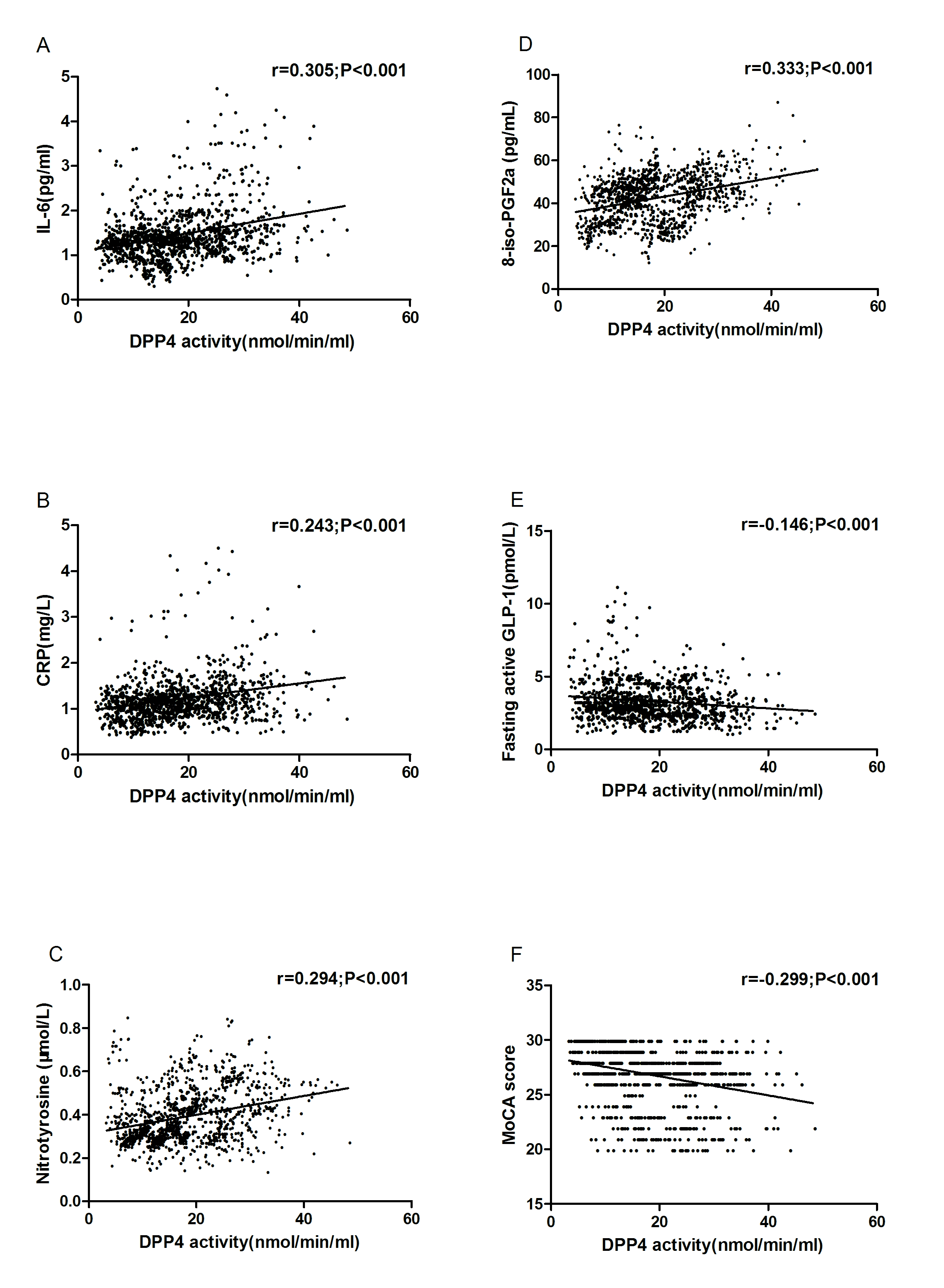

Supplement: FIGURE S1 — Correlations between DPP4 activities vs metabolic parameters and MoCA score represented by scatter plot. (A) relationship between DPP4 activity and IL-6; (B) relationship between DPP4 activity and CRP; (C) relationship between DPP4 activity and nitrotyrosine; (D) relationship between DPP4 activity and 8-iso-PGF2a; (E) relationship between DPP4 activity and fasting active GLP-1; (F) relationship between DPP4 activity and MoCA score. [file Image_1.TIF]
