# Supplementary material for: Strong Association between Plasma Dipeptidyl Peptidase-4 Activity and Impaired Cognitive Function in Elderly Population with Normal Glucose Tolerance
Source: Front Aging Neurosci. 2017 Jul 26;9:247. doi: 10.3389/fnagi.2017.00247 (PMC5526854; doi:10.3389/fnagi.2017.00247)
Supplement: Supplementary file 2 [file Table_1.DOC]

Supplementary table1. Logistic regression analysis of the association between DPP4 activity and MCI in a subgroup of participants without hyperuricemia and hypertriglyceridemia

|  | DPP4 activity | | | |
| --- | --- | --- | --- | --- |
|  | Q1 | Q2 | Q3 | Q4 |
| DPP4 activity  (nmol/ml/min) | <11.5 | 11.5-16.7 | 16.8-23.9 | >23.9 |
| MCI | 23(9.0%) | 39(15.3%) | 41(16.1%) | 65(25.7%) |
| Model 1 | 1 | 1.82(1.05, 3.15) 0.032 | 1.93(1.12, 3.33) 0.017 | 3.49(2.09, 5.83) <0.001 |
| Model 2 | 1 | 1.74(0.99, 3.05) 0.051 | 1.88(1.08, 3.28) 0.026 | 3.16(1.87, 5.34) <0.001 |
| Model 3 | 1 | 1.71(0.98, 2.99) 0.061 | 1.92(1.10, 3.36) 0.022 | 3.31(1.93, 5.67) <0.001 |
| Model 4 | 1 | 1.74(0.99, 3.05) 0.051 | 1.88(1.08, 3.29) 0.027 | 3.16(1.86, 5.36) <0.001 |
| Model 5 | 1 | 1.48(0.84, 2.61) 0.175 | 1.78(1.01, 3.12) 0.045 | 2.30(1.32, 4.00) 0.003 |

Model1: crude model

Model2: Model1+ age + gender + BMI + current smoking + habitual alcohol consumption + leisure-time physical activity + education level + annual income + cardiovascular disease + NSAID use + SBP

Model3: Model2 + IL-6

Model4: Model2 + fasting active GLP-1

Model5: Model2 + 8-iso-PGF2a
